# Methodological issues in detecting gene-gene interactions in breast cancer susceptibility: a population-based study in Ontario

**DOI:** 10.1186/1741-7015-5-22

**Published:** 2007-08-07

**Authors:** Laurent Briollais, Yuanyuan Wang, Isaac Rajendram, Venus Onay, Ellen Shi, Julia Knight, Hilmi Ozcelik

**Affiliations:** 1 Prosserman Centre for Health Research, Samuel Lunenfeld Research Institute, Mount Sinai Hospital, Toronto, M5T 3L9, Canada; 2 Public Health Sciences Department, University of Toronto, Toronto, M5T 3M7, Canada; 3 Fred A Litwin Centre for Cancer Genetics, Samuel Lunenfeld Research Institute, Mount Sinai Hospital, Toronto, M5T 3L9, Canada; 4 Department of Pathology and Laboratory Medicine, Mount Sinai Hospital, Toronto, M5G 1X5, Canada; 5 Ontario Cancer Genetics Network, Cancer Care Ontario, Toronto, M5G 2L9, Canada

## Abstract

**Background:**

There is growing evidence that gene-gene interactions are ubiquitous in determining the susceptibility to common human diseases. The investigation of such gene-gene interactions presents new statistical challenges for studies with relatively small sample sizes as the number of potential interactions in the genome can be large. Breast cancer provides a useful paradigm to study genetically complex diseases because commonly occurring single nucleotide polymorphisms (SNPs) may additively or synergistically disturb the system-wide communication of the cellular processes leading to cancer development.

**Methods:**

In this study, we systematically studied SNP-SNP interactions among 19 SNPs from 18 key genes involved in major cancer pathways in a sample of 398 breast cancer cases and 372 controls from Ontario. We discuss the methodological issues associated with the detection of SNP-SNP interactions in this dataset by applying and comparing three commonly used methods: the logistic regression model, classification and regression trees (CART), and the multifactor dimensionality reduction (MDR) method.

**Results:**

Our analyses show evidence for several simple (two-way) and complex (multi-way) SNP-SNP interactions associated with breast cancer. For example, all three methods identified *XPD*-[Lys751Gln]*IL10-[G(-1082)A] as the most significant two-way interaction. CART and MDR identified the same critical SNPs participating in complex interactions. Our results suggest that the use of multiple statistical approaches (or an integrated approach) rather than a single methodology could be the best strategy to elucidate complex gene interactions that have generally very different patterns.

**Conclusion:**

The strategy used here has the potential to identify complex biological relationships among breast cancer genes and processes. This will lead to the discovery of novel biological information, which will improve breast cancer risk management.

## Background

A grand challenge in statistical genetics is to develop powerful methods that can identify genes that control biological pathways leading to disease. Discovery of such genes is critical in the detection and treatment of human diseases, including cancer. The dramatic advances in human genome research coupled with the recent progress in high-throughput technology for molecular biology and genetics now allow the study of the genetic basis of disease and the response to treatment of complex diseases, such as breast cancer, on a molecular level. A good example is the recent efforts of the Human Genome Project towards large-scale characterization of human single nucleotide polymorphisms (SNPs). SNPs are an abundant form of genomic variation, distinguished from rare variations by the requirement that the least abundant allele have a frequency of 1% or more. In view of the fact that genetic and phenotypic variability exist among individuals, it has long been hypothesized that an individual's SNP pattern has significant impact on their susceptibility to disease, and their response to therapies [1]. One of the main challenges in searching for disease alleles is to understand how and when particular genetic variants or combinations of variants are associated with disease. Indeed, most complex human diseases result from the poorly understood interaction of genetic and environmental factors. The genetic determinants of such diseases result, in turn, from the poorly understood interaction of dozens, if not hundreds, of disease genes. Breast cancer provides a useful paradigm for studying genetically complex diseases. The existence of dominant predisposition genes conferring a high breast cancer risk has been confirmed with the discovery of *BRCA1 *[2] and *BRCA2 *[3,4] genes. Mutations in these genes dramatically alter the function of encoded proteins in breast cells, leading to tumor formation. These functionally effective mutations are strongly associated with dramatically increased breast cancer risk. However, mutant alleles of such highly penetrant genes are relatively rare (<5%) in unselected breast cancer cases. Besides these alleles, many low penetrant variants, mostly due to SNPs, also contribute to breast cancer risk [5-7]. In contrast to highly penetrant mutations, commonly occurring SNPs are usually associated with less dramatic effects on the function of encoded proteins and thus, individually, they contribute incrementally to cumulative breast cancer risk. Nonetheless, inheritance of combinations of functional and cancer-linked commonly occurring SNPs may additively or synergistically disturb the system-wide communication of the biological processes, leading to cancer. Therefore, individuals carrying several interacting susceptibility alleles could have a high risk of developing breast cancer.

In this present study, we assessed the importance of SNP-SNP interactions on breast cancer risk by investigating 19 SNPs from genes involved in major cancer related pathways and biological systems in a sample of 398 breast cancer cases and 372 healthy population controls. We compared and evaluated three methods to detect simple and complex SNP-SNP interactions: the logistic regression model (LRM), the classification and regression trees (CART), and the multifactor dimensionality reduction (MDR) approach. Model validation and permutation tests are also proposed along with these methods to decrease the rate of false positive results and to provide a distribution-free test statistic. By comparing results from these three methods, we identified interesting genetic interaction patterns and discuss the pros and cons of each method in studying SNP-SNP interactions.

## Materials and methods

### Subject population

A case control study was conducted using biospecimens and data from the Ontario Familial Breast Cancer Registry (OFBCR), a participating site in the NIH-funded Breast Cancer Family Registry [8]. Written informed consent was obtained from all subjects, and the study protocol was approved by the Mount Sinai Hospital Research Ethics Board. Cases of invasive breast cancer, pathologically confirmed and diagnosed between 1996 and 1998 in the province of Ontario, were identified from the population-based Ontario Cancer Registry. All female cases under the age of 55 years were identified as well as all males under the age of 80 years. A random sample (35%) of female cases aged 55 to 69 was also selected. Physician permission to contact patients was granted for 91% of the cases (7,668 of 8,453). Patients were then mailed a cancer family history questionnaire and 65% (4,957) of them completed it. All respondents who met a defined set of genetic risk criteria (that is, Ashkenazi Jewish; diagnosed before age 36 years; previous ovarian or breast diagnosis; one or more first- or two or more second-degree relatives with breast or ovarian cancer; one or more second- or third-degree relatives with either breast cancer diagnosed before age 36 years, ovarian cancer diagnosed before age 61 years, multiple breast or breast and ovarian primaries, or male breast cancer; three or more first-degree relatives with any combination of breast, ovarian, colon, prostate, or pancreatic cancer or sarcoma, with at least one diagnosis before age 51 years) were included in the study [9] and a random sample of 25% of those not meeting these criteria were selected to continue to participate in the OFBCR (n = 2,580). This participation included providing a blood sample, which was provided by 62% of all eligible participants (n = 1,601). For the current study, we restricted the sample to women who identified themselves as Caucasian and were less than 55 years old. The study was restricted to the younger women because they are more likely to have genetic causes. Women in this age group might also be more homogeneous regarding their breast cancer risk factors. As we had randomly sampled 25% of those who did not meet genetic risk criteria, we also randomly sampled 25% of those who did meet genetic risk criteria in order to create a more representative sample of cases. Therefore, the selected cases should better represent all cases without enrichment for genetic risk criteria such as family history. A total of 21.6% of cases in the present study had a first-degree family history of breast cancer, which is consistent with the 17–22% frequency reported in cases in a number of large case-control studies [10-12]. Of the 459 breast cancer cases with an available blood sample, 398 were successfully genotyped and included in this study after excluding cases with insufficient DNA and those who were not Caucasians.

Controls were identified by calling randomly selected residential telephone numbers from across the province of Ontario and were frequency-matched to all female OFBCR cases by five-year age group. The number of telephone numbers was 14,653, but 1,101 (8%) were invalid and no contact could be made for 841 (6%). Of the 12,711 households contacted, 7,829 (62%) did not have an eligible individual. No information on eligibility was provided for 2,194 (17%) households. Of the 2,688 eligible individuals identified on the telephone, 1,726 (64%) completed the mailed risk factor questionnaire and 75% of these individuals agreed to be contacted about providing a blood sample. Information regarding past history of cancer and family history of cancer was collected on the controls. The 676 women under age 55 who had agreed to be approached about blood sampling were asked to provide a blood sample and 419 (62%) did so. Individuals who were not Caucasian were excluded from the analysis, as were those with insufficient DNA or those subsequently found to be ineligible because of age. The remaining 372 population controls were successfully genotyped for this study.

### SNP selection strategy

To estimate the breast cancer risk conferred by individual SNPs, as well SNP-SNP interactions, we studied 19 SNPs from 18 key cancer genes involved in several biological pathways. Candidate SNPs were initially selected using the best available evidence from published studies at the beginning of the project in 2000 and were subsequently classified into three categories (high, medium and low rank). Thus, they include SNPs with a wide range of functional evidence. High-ranking SNPs were supported by studies that demonstrated the effect of the SNP on the regulation of expression or protein function. The medium-rank category is likely to include functionally relevant SNPs, as the substitutions are predicted to significantly affect function, although this was not confirmed experimentally at the time of their selection. This category also includes SNPs that are associated with breast cancer risk factors. The low ranking category, on the other hand, contained SNPs with no functional information. Among the SNPs studied, *XPD*-[Lys751Gln], *MTHFR*-[Ala222Val], *COMT*-[Met108/158Val], *GSTP1*-[Ile105Val] and *CCND1*-[Pro241Pro] have been shown to alter the function or post-translational modification of their encoded protein [13-25]. *MMP1*-[1G(-1607)2G] and *IL10*-[G(-1082)A] have been shown to alter the transcription and expression of these genes [26-30]. *IL13*-[Arg130Gln] has been suggested to have functional consequences, while GSTM3-[4595 (3 bp ins/del)] was predicted to create a YY1 transcription factor binding site [31,32]. *TNFA*-[G(-308)A] forms a haplotype with some nearby SNPs and some studies observed increased haplotype-dependent transcriptional activity change while some others did not [33-36]. *CYP17*-[C518T] and *IL13*-[Arg130Gln] were found to be associated with other cancer-related variables, such as serum estrogen and IgE levels, respectively [37-39]. *BARD1*-[Pro24Ser] changes a structurally important non-polar proline residue to a positively charged serine. There were no functional speculations for *ESR1*-[Ser10Ser], *ESR1*-[Pro325Pro], *PTEN*-[(IVS4+109)ins/delACTAA], *IL1A*-[Ala114Ser], *G-CSF*-[Leu185Leu] and *GADD45*-[C(IVS3+168)T. Thus, the 19 SNPs studied represent SNPs with a wide range of functional knowledge and evidence. The estimate of the minor allele frequency for the 19 SNPs studied varies between 15% and 48% in the general population. The SNPs studied were also selected to represent more commonly occurring variants, in order to gain statistical power to detect SNP-SNP interactions.

### Molecular genotyping

All SNPs were analyzed by TaqMan 5' nuclease assay [40] using the 7900 HT Sequence Detection System (ABI, Foster City, CA, U.S.A.). Oligonucleotide primers and the dual labeled allele specific probes were designed using Primer Express version 2.0 (PE Biosystems, Foster City, CA, U.S.A.). Positions of primers and probes and their appropriate accession numbers are given in Additional data file 1. A panel of DNA samples was sequenced for each SNP region initially, in order to identify control genotypes to be used in each experiment. PCRs were performed in 96-well plates (AXYGEN, Union City, CA, U.S.A.), with each plate containing four control samples for each possible genotype. Genomic DNA (10 ng) was amplified in a total volume of 10 μl in the presence of 100 μM of each of the dNTPs, 3 pmoles of each of the appropriate primers, 2 pmoles of each of the corresponding dual labeled probes, and 0.025 units of Platinum Taq DNA Polymerase (Invitrogen, Carlsbad, CA, U.S.A.). PCR cycling conditions consisted of 40 cycles of 94°C for 15 s, 55–60°C for 15 s and 72°C for 15 s. The samples were analyzed by ABI PRISM 7900 HT Sequence Detection System (version 2.0). The optimal MgCl_2 _concentrations and annealing temperatures for each SNP are given in Additional data file 2. The results were validated by re-genotyping a randomly selected 10% portion of the total study population [41].

### Statistical analysis

To detect complex interactions, we applied and compared three statistical methods, LRM, CART and MDR. Our main analyses are presented without adjustment for other confounding variables. However, we also conducted several analyses adjusted for age, family history and body mass index (BMI) and our results were quite similar to those presented here. Our dataset included five individuals with missing genotypes, so their missing values were randomly imputed from the empirical distribution of the genotypes in the sample. We also carried out several analyses without these individuals and our results remained unchanged. In all of our analyses, the SNP variables were considered as nominal categorical variables with three categories.

### Logistic regression model

LRM is a parametric approach that relates one or more explanatory variables, Xs (for example, SNPs and their interactions), to a dependent or outcome variable, Y (for example, breast cancer status). LRM is a particular case in the family of generalized linear models [42]. The simple LRM for an individual *i *assumes that *Y*_*i *_takes values 0 or 1 and has a Bernoulli distribution with parameter *P*_*i*_, that is, the probability of being a case rather than a control: *Pr*(Y_i _= 1) = *P*_*i*_, and *Pr*(Y_i _= 0) = 1 - *P*_*i*_. It is expressed as:

LogitPi=log⁡Pi1−Pi=β0+∑j=1pβjxj+∑j=1p−1∑k>jpβjkxjxk,
 MathType@MTEF@5@5@+=feaafiart1ev1aaatCvAUfKttLearuWrP9MDH5MBPbIqV92AaeXatLxBI9gBaebbnrfifHhDYfgasaacH8akY=wiFfYdH8Gipec8Eeeu0xXdbba9frFj0=OqFfea0dXdd9vqai=hGuQ8kuc9pgc9s8qqaq=dirpe0xb9q8qiLsFr0=vr0=vr0dc8meaabaqaciaacaGaaeqabaqabeGadaaakeaacqWGmbatcqWGVbWBcqWGNbWzcqWGPbqAcqWG0baDcqWGqbaudaWgaaWcbaGaemyAaKgabeaakiabg2da9iGbcYgaSjabc+gaVjabcEgaNnaalaaabaGaemiuaa1aaSbaaSqaaiabdMgaPbqabaaakeaacqaIXaqmcqGHsislcqWGqbaudaWgaaWcbaGaemyAaKgabeaaaaGccqGH9aqpiiGacqWFYoGydaWgaaWcbaGaeGimaadabeaakiabgUcaRmaaqahabaGae8NSdi2aaSbaaSqaaiabdQgaQbqabaGccqWG4baEdaWgaaWcbaGaemOAaOgabeaakiabgUcaRmaaqahabaWaaabCaeaacqWFYoGydaWgaaWcbaGaemOAaOMaem4AaSgabeaakiabdIha4naaBaaaleaacqWGQbGAaeqaaOGaemiEaG3aaSbaaSqaaiabdUgaRbqabaaabaGaem4AaSMaeyOpa4JaemOAaOgabaGaemiCaahaniabggHiLdaaleaacqWGQbGAcqGH9aqpcqaIXaqmaeaacqWGWbaCcqGHsislcqaIXaqma0GaeyyeIuoakiabcYcaSaWcbaGaemOAaOMaeyypa0JaeGymaedabaGaemiCaahaniabggHiLdaaaa@712C@

where *i *= *1,..,n*, and *p *is the number of explanatory variables. The *βs *are the regression coefficients that are to be estimated: *β*_*j *_for the SNP main effects (*x*_*j*_) and *β*_*jk *_for the SNP-SNP interaction (*x*_*j*_*x*_*k*_). Because the genetic risk model is unknown for most of the SNPs we studied, we adopted a codominant model, that is, both rare homozygous and heterozygous variant effects are estimated using two dummy variables for the SNP main effects and four product terms for the two-way interaction effects. Interaction effects were tested using a likelihood ratio test (LRT) statistic with four degrees of freedom for the *χ*^2 ^values. We used a stepwise logistic-regression procedure based on forward selection to select the most significant SNP-SNP interactions [43]. Forward stepwise selection procedures are efficient in assessing interaction effects compared to backward elimination when testing multiple interactions. First, it is more computationally efficient and second, compared to backward elimination where a relatively large number of predictor variables may increase the risk of complete separation of the two outcome groups, it has fewer numerical problems when estimating the model parameters [44]. We tested only two-way interactions with LRM since the investigation of higher-order interactions could have led to numerical difficulties for estimating the model parameters. Instead of using a stepwise selection procedure, one could perform an exhaustive search and select the most significant interactions based on a permutation test or using the false discovery rate (FDR) principle [41]. This was not done in this study because this strategy is not directly applicable to CART analyses.

### Classification and regression trees

**Figure 1:**
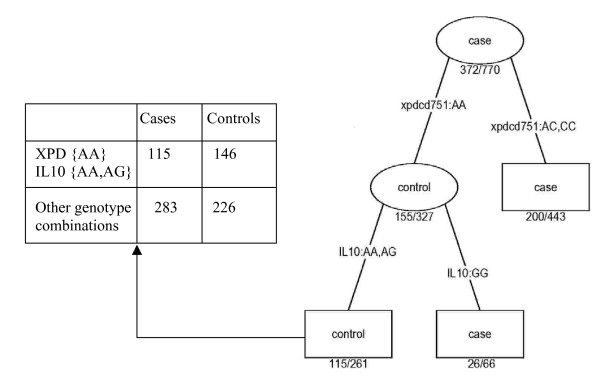
Application of CART to the XPD*IL10 interaction. CART sequentially partitions the data into two homogeneous subsets: first using *XPD*-[Lys751Gln], {AA} versus {AC, CC}; and then the {AA} subset is split according to *IL10*-[G(-1082)A], {AA, AG} versus {GG}. The splitting variables leading to such groups are inherent main or interaction effects. For example, a low-risk subgroup is defined by the two SNPs: *XPD*-[Lys751Gln] and *IL10*-[G(-1082)A] and the tree suggests an interaction between these two SNPs. Multi-way interactions can be detected in a similar way. The terminal nodes can be classified as low- or high-risk subgroups (indicated by different color density) and their association with the outcome can be estimated (that is, the corresponding odds ratio is 0.63 with a *P*-value of 0.002). Therefore, investigating the tree terminal nodes provides a natural way to identify interactions and characterize high- or low-risk subgroups.

Decision trees date back to the early 1960s with the work of Morgan and Sonquist [45]. Breiman and colleagues [46] published the first comprehensive description of recursive partitioning methodology. As a powerful data analysis method, trees are used in many fields, such as epidemiology and medical diagnosis, and provide an alternative to more standard model-based regression techniques for multivariate analyses. The tree introduced here is the *S *implementation [47]. Because our outcome (case or control status) is binary, we used classification trees. Through binary recursive partitioning, a tree successively splits the data along the coordinate axes of the predictors such that, at each division, the resulting two subsets of data are as homogeneous as possible with respect to the response of interest [46]. At each step in the construction algorithm, an optimal split is identified. This local optimality does not guarantee that the overall optimal tree will be found. Deviance is a natural splitting criterion based on likelihood values. In our analyses, we used the *S *defaults: a node must include at least 10 observations and the minimum node deviance before the tree growing stops should be 1% of the root node. The subsets that are not further split are the terminal nodes. The SNP variables were considered as nominal categorical variables with three categories. Figure 1 depicts the first few nodes of a tree built on our data and illustrates how a tree is used for prediction.

### Multifactor dimensionality reduction

MDR [48-51] is a non-parametric data mining approach that uses constructive induction or attribute construction to reduce two or more SNPs, for example, to a new single variable that is then evaluated using a classifier such as naive Bayes or logistic regression. The rationale behind this method is to identify the multi-locus genotypes that best predict the outcome of interest. It applies data reduction techniques to address problems associated with testing for interactions in high-dimensional space and with generally modest sample sizes. The algorithm works as follows.

First, select all subsets of *k *explanatory variables (that is, SNPs) among *m *available and for each subset *k*, enumerate all possible genotype combinations. These genotype combinations represent the interacting SNPs. For each combination, compute the case-control ratio and partition the multi-locus genotypes into two subgroups labeled as high or low risk (for example, ratio ≥1 or ratio <1). This step reduces a *k*-dimensional model to one dimension only.

Second, a ten-fold cross-validation (CV) procedure is used to assess the ability of the multi-locus genotype combinations to predict the disease outcome. The sample is divided into a training set (for example, 9/10 of cases and controls) and an independent test set (for example, 1/10 of cases and controls). In each subset of the data, the training set classification error is computed for each genotype combination. This step is repeated in the ten random subsets. The interaction with the lowest classification error (averaged over the ten random subsets) is selected as the 'best interaction'. The whole CV procedure is itself repeated ten times to protect against chance division and all resulting statistics are averages over these ten runs.

Third, all interactions selected through steps 1 and 2 are also evaluated for their CV consistency (number of times the same genotype combination is identified across the ten repetitions of the CV procedure) and testing accuracy (that is, 1 - prediction error).

Fourth, steps 1 through 3 are repeated for different values of *k *(2, 3, 4, 5 and 6 in our study). The interaction that maximizes the CV consistency and testing accuracy is selected as the final best candidate interaction across all *k*-multilocus models. In our study, instead of selecting just one best interaction for each *k*, as proposed originally for this method, we selected the five best interactions based on the same criteria.

### Modeling and interpreting SNP-SNP interactions

**Figure 2:**
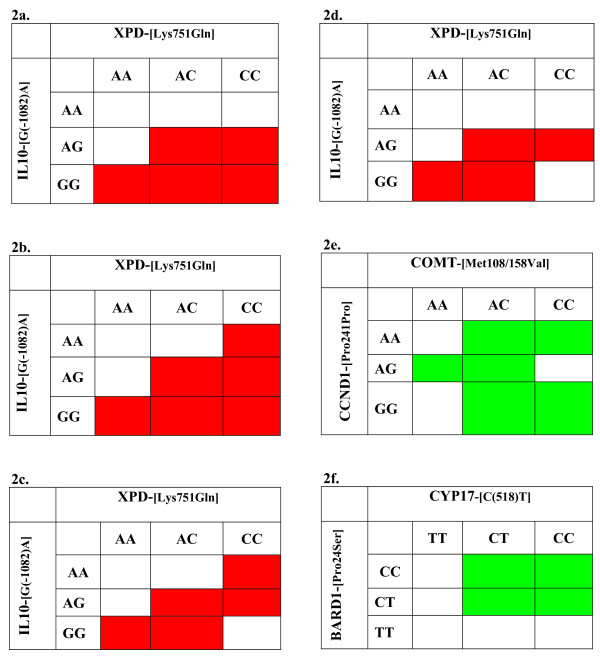
**Example of partitions of two-locus genotypes with the three methods**. **(a-d) **The four partitions identified by MDR for the *XPD-CYP17 *two-locus genotypes. **(e) **The best partition found by MDR for the *COMT-CCDN1 *two-locus genotypes. **(f) **The best partition found by CART for the *CYP17-BARD1 *two-locus genotypes. Shaded cells are classified as high-risk and non-shaded cells as low-risk. This corresponds to a ratio of cases versus controls higher or lower than 1, respectively. The four partitions of the two-locus genotypes found by MDR showed two cells with different assignments. In (f), CART can partition the two-locus genotypes in more than two groups, but for the purpose of comparison with MDR, we used the same high-risk/low-risk grouping.

With LRM, interactions are taken into account by estimating specific parameters for certain genotype combinations. Assuming a codominant effect for each SNP, the LRM is a saturated model with nine parameters coding for the cell-specific odds-ratios ((exp(*β*_*j*_), equation 1). LRM estimates the cell-specific odds-ratio corresponding to each genotype combination. A saturated model tends to fit the observed data but could lead to overfitting and a lack of power. CART suggests interactions by the successive splits occurring in the tree (Figure 1). The terminal nodes correspond to either low- or high-risk subgroups and their association with the outcome can be tested. Investigating the tree terminal nodes provides a natural way to identify interaction and characterize the high- or low-risk subgroups. Moreover, CART automatically selects the genetic model that best fits the data. A fundamental difference between CART and LRM is that CART can reveal the structure in the data by creating a split in the proportion of the data only where it is pertinent (for example, among individuals who have the *XPD *{AA} genotype in Figure 1). LRM does not have this feature and can test only an overall effect. MDR, like CART, partitions the data into high- and low-risk subgroups based on the SNP profiles. However, while CART uses recursive partitioning to reveal the interactions, MDR exhaustively searches across all multi-locus genotypes and identifies the one that best predicts the disease outcome. A particular aspect of MDR is that each selected interaction could correspond to different genotype combinations when repeating the CV procedure. For example, Figure 2a–d displays the four partitions corresponding to the *XPD*-[Lys751Gln] – *IL10*-[G(-1082)A] two-locus genotypes when repeating the 10-fold CV procedure ten times.

### Strategy to identify critical SNP-SNP interactions associated with breast cancer

All three methods were applied to our data to detect two-way interactions; CART and MDR were also used to investigate higher-order interactions. Because MDR uses a specific strategy to select the important interactions, we tried to develop a similar approach for LRM and CART, which can be summarized by two major steps: first, selection of the most promising SNP-SNP interactions using a CV approach; and second, evaluation of the selected interactions in the whole sample using a distribution-free permutation test.

#### Step 1

With LRM and CART, we used a two-fold CV approach to select the SNP-SNP interactions, instead of the ten-fold CV of MDR. Our rationale is that a test statistic is used to select the best interactions with LRM and CART, and splitting the original sample into two ensures enough power to perform this test. The idea is to randomly divide the original dataset into training and test sets, each with the same proportion of cases and controls. A variable selection of the important SNP-SNP interactions is carried out in the training set and their significance is evaluated independently in the test set. With LRM, we used forward stepwise selection with the training set and then tested each selected interaction using a LRT statistic in the test set. With CART, we built a tree on the training set, and pruned it to a smaller tree using the deviance criteria in order to keep a maximum of ten terminal nodes. We then applied the pruned tree on the test set and calculated a chi-square statistic for each terminal node. To protect against chance divisions of the data, the CV is repeated ten times and the test statistic for each selected interaction in the test set is averaged over the ten random test sets. The five best two-way interactions (terminal nodes with the larger test statistic) are then selected. With CART and MDR, we also selected the top five *k*-way interactions with *k = *3,4,5,6.

#### Step 2

We then evaluated each target (that is, selected interaction) in the whole sample using a permutation test statistic. With LRM, we compared a model with and without the selected interaction using the original dataset (main effects are also in the model) and obtained the chi-square statistic from the LRT (each test had four degrees of freedom). With CART, we collected the chi-square statistic for each terminal node and computed the odds-ratio associated with the corresponding subgroup on the original dataset (see Figure 1 for an example). With MDR, we collected the testing accuracy for each target, averaged across the 100 random subsets (that is 10-fold CV repeated 10 times). To find the null distribution of the test statistics, we randomly permuted the response variable 1,000 times. The *P*-value associated with each target is then computed by comparing the observed test statistic to its empirical null distribution. The null hypothesis was rejected when the upper-tail Monte-Carlo *P*-value derived from the permutation test is less than 5%.

### Software

We used the Splus package 'tree' (Insightful Corporation, Seattle, WA) and some related Splus functions for the CART analyses and the R package 'glm' [52] for the LRM analyses. MDR analyses were carryout with the program MDR v.1.4.1 for Unix [48-51].

## Results

### Individual SNP effects

Among the 19 SNPs studied, *XPD*-[Lys751Gln] was the only one showing a significant main effect in our sample based on the crude *P-*value from LRM. However, after correction for multiple testing using the FDR principle [53], the effect was not significant. Our results remained unchanged when the models were also adjusted for age, BMI and family history. A more comprehensive description of the analysis of the SNP main effects and the baseline characteristics of breast cancer cases and controls can be found in [41].

### Evaluation of two-way SNP-SNP interactions

All three methods declared *XPD*-[Lys751Gln]*IL10-[G(-1082)A] as the most significant two-way interaction (Table 1). MDR and LRM further identified *COMT*-[Met108/158Val]*CCND1-[Pro241Pro] as the second most significant interaction, while CART selected *BARD1*-[Pro24Ser]*CYP17-[C(518)T]. However, there were also some discrepancies across the three methods, which we detail below.

**Table 1:** Two-way interactions detected by the three methods with a *P*-value less than 5%

Rank	LRM		CART		MDR	
			
	Interaction	*P*-value	Interaction	*P*-value	Interaction	*P*-value
1	*XPD*-[Lys751Gln] *IL10*-[G(-1082)A]	0.013	*XPD*-[Lys751Gln] *IL10*-[G(-1082)A]	0.006*	*XPD*-[Lys751Gln] *IL10*-[G(-1082)A]	0.009*
2	*COMT*-[Met108/158Val] *CCND1*-[Pro241Pro]	0.020	*CYP17*-[C(518)T] *BARD1*-[Pro24Ser]	0.013	*COMT*-[Met108/158Val] *CCND1*-[Pro241Pro]	0.019
3	*TNFA*-[G(-308)A] *p27*-[Val109Gly]	0.046			*BARD1*-[Pro24Ser] *XPD*-[Lys751Gln]	0.037
4					*IL13*-[Arg130Gln] *CYP17*-[C(518)T]	0.037

*Significant after Bonferroni correction for the number of tests performed in the second stage analysis.

Three two-way interactions found by MDR, *COMT*-[Met108/158Val]*CCND1-[Pro241Pro], *BARD1*-[Pro24Ser]*XPD-[Lys751Gln] and *IL13*-[Arg130Gln]*CYP17-[C(518)T], were not detected by CART. The patterns of these interactions are all complex. For example, the interaction *COMT*-[Met108/158Val]*CCND1-[Pro241Pro] seems to involve a codominant effect for *COMT*-[Met108/158Val] and an interference effect for *CCND1*-[Pro241Pro] [54] (Figure 2b). To model this interaction, CART would have to split the data first according to *COMT*-[Met108/158Val] into {AA} and {AG, GG} and then the subset {AG, GG} into {AG} and {GG}. The three nodes should then be split into two subsets according to *CCND1*-[Pro241Pro] genotypes. This type of structure is not easily captured by CART [55]. Another explanation is that interactions not involving *XPD*-[Lys751Gln] and *CYP17*-[C(518)T], the two most significant main effects, are difficult to detect by CART. Because of the binary splits, CART is more likely to detect interactions in the presence of a strong main effect.

One two-way interaction found by CART, *BARD1*-[Pro24Ser]*CYP17-[C(518)T], was not detected by MDR. The two-way contingency table corresponding to this interaction exhibits many cells with a case-control ratio close to 1.0. This means that many individuals can be classified as either high or low risk, so the testing accuracy is low (that is, prediction error is high). On the other hand, CART can easily identify this interaction, first assuming a dominant effect for *CYP17*-[C(518)T] (that is, {TT} and {CT, CC}), and then a recessive effect for *BARD1*-[Pro24Ser] (that is, {CC, CT} and {TT}) (Figure 2c). The binary splits provided by CART coincide with simple genetic models for each SNP (dominant or recessive).

Three two-way interactions found by either MDR or CART are not detected by LRM. Unlike CART and MDR, LRM does not partition the data but uses a global test of interaction based on all individuals. This test will be powerful if the global chi-square has support throughout the nine two-locus genotype combinations. See the interaction between *TNFA*-[G(-308)A] and *p27*-[Val109Gly], for example (Table 1). LRM is less sensitive to detecting local effects. CART finds the partition that maximizes the chi-square for a two-group comparison. MDR finds genotype combinations that maximize the testing accuracy (1 - prediction error) of the disease outcome. These two methods have power to find interactions if there is good discrimination between the low- and high-risk subgroups; they can both detect local effects too.

### Evaluation of higher-order interactions

The interactions detected by CART and MDR are listed in Tables 2 and 3, respectively. A total of seven interactions were detected by CART, including five high-order interactions (three-way or higher). These interactions involve 11 SNPs out of the 19 SNPs available. *XPD*-[Lys751Gln], the only significant main effect, appears in four out of the seven interactions. With CART, many risk subgroups are nested within each other. Therefore, there is some redundancy in the patterns of CART interactions. Interestingly, most interactions can be described as simple combinations of dominant and recessive genetic models for the effect of each individual SNP. There are also a few interactions involving interference effects (that is, neither dominant nor recessive). The patterns of interactions detected by CART have, in general, an easy interpretation. MDR interactions involve 14 SNPs, including the 11 SNPs suggested by CART. Therefore, the interactions found by MDR are slightly more diverse than those detected by CART. However, both methods seem to agree on the strongest SNP effects contributing to breast cancer risk. *XPD*-[Lys751Gln], the only significant main effect, appears in 12 interactions in the MDR approach. The two most significant two-way interactions, *XPD*-[Lys751Gln]*IL10-[G(-1082)A] and *COMT*-[Met108/158Val]*CCND1-[Pro241Pro], are found nine and seven times, respectively. Similar to CART, there is also some redundancy in the patterns of MDR results. The testing accuracies calculated by MDR ranges from 60.2% to 52.4%, which is a modest improvement over the rate of 50% expected under random prediction. The risk subgroups suggested by MDR are, in general, very complex but their interpretation can be facilitated by the use of an entropy-based interaction dendrogram [51].

**Table 2:** Interactions and risk subgroups identified by CART with a *P*-value less than 5%

Rank	Interaction	*P*-value
1	*XPD*-[Lys751Gln] *IL10*-[G(-1082)A]	0.006
2	*CYP17*-[C(518)T] *BARD1*-[Pro24Ser]	0.013
3	*XPD*-[Lys751Gln] *IL10*-[G(-1082)A] *IL1A*-[Ala114Ser] *ESR1*-[Ser10Ser]	0.016
4	*CYP17*-[C(518)T] *IL13*-[Arg130Gln] *IL1A*-[Ala114Ser] *MTHFR*-[Ala222Val] *PTEN-*[(IVS4+109)ins/del5	0.019
5	*XPD*-[Lys751Gln] *BARD1*-[Pro24Ser] *MTHFR*-[Ala222Val]	0.020
6	*CYP17*-[C(518)T] *BARD1*-[Pro24Ser] *MTHFR*-[Ala222Val]	0.043
7	*XPD*-[Lys751Gln] *GADD45*-[C(IVS3+168)T] *GSTP1*-[Ile105Val]	0.050

**Table 3:** Interactions selected by MDR with permutation *P*-value less than 5%

Rank	Interactions	Testing accuracy*	Permutation *P*-value
1	*IL1A*-[Ala114Ser] *XPD*-[Lys751Gln] *IL10*-[G(-1082)A]	58.2%	<0.001
2	*ESR1*-[Ser10Ser] *CYP17*-[C(518)T] *IL10*-[G(-1082)A] *COMT*-[Met108/158Val] *PTEN- *[(IVS4+109)ins/del5] *CCND1*-[Pro241Pro]	60.2%	<0.001
3	*CYP17*-[C(518)T] *BARD1*-[Pro24Ser] *COMT*-[Met108/158Val] *MMP1*-[1G(-1607)2G] *CCND1*-[Pro241Pro]	58.4%	0.001
4	*IL13*-[Arg130Gln] *XPD*-[Lys751Gln] *IL10*-[G(-1082)A]	57.5%	0.002
5	*CYP17*-[C(518)T] *XPD*-[Lys751Gln] *GSTP1*-[Ile105Val] *GADD45*-[C(IVS3+168)T]	56.7%	0.006
6	*XPD*-[Lys751Gln] *IL10*-[G(-1082)A] *GSTP1*-[Ile105Val] *COMT*-[Met108/158Val] *MMP1*-[1G(-1607)2G] *CCND1*-[Pro241Pro]	57.5%	0.006
7	*ESR1*-[Ser10Ser] *XPD*-[Lys751Gln] *IL10*-[G(-1082)A] *MMP1*-[1G(-1607)2G] *PTEN*-[(IVS4+109)ins/del5	57.2%	0.007
8	*CYP17*-[C(518)T] *XPD*-[Lys751Gln] *IL10*-[G(-1082)A] *MTHFR*-[Ala222Val] *COMT*-[Met108/158Val] *CCND1*-[Pro241Pro]	58.2%	0.007
9	*XPD*-[Lys751Gln] *IL10*-[G(-1082)A]	55.9%	0.009
10	*CYP17*-[C(518)T] *XPD*-[Lys751Gln] *IL10*-[G(-1082)A] *MTHFR*-[Ala222Val] *COMT*-[Met108/158Val]	56.3%	0.016
11	*BARD1*-[Pro24Ser] *XPD*-[Lys751Gln] *IL10*-[G(-1082)A]	55.6%	0.017
12	*ESR1*-[Ser10Ser] *XPD*-[Lys751Gln] *MMP1*-[1G(-1607)2G] *PTEN*-[(IVS4+109)ins/del5	55.9%	0.017
13	*COMT*-[Met108/158Val] *CCND1*-[Pro241Pro]	55.2%	0.019
14	*CYP17*-[C(518)T] *IL10*-[G(-1082)A] *COMT*-[Met108/158Val] *PTEN*-[(IVS4+109)ins/del5] *CCND1*-[Pro241Pro]	55.7%	0.020
15	*IL10*-[G(-1082)A] *MTHFR*-[Ala222Val] *GSTP1*-[Ile105Val] *COMT*-[Met108/158Val]	55.7%	0.023
16	*IL13*-[Arg130Gln] *CYP17*-[C(518)T]	54.5%	0.037
17	*BARD1*-[Pro24Ser] *XPD*-[Lys751Gln]	54.7%	0.037
18	*CYP17*-[C(518)T] *XPD*-[Lys751Gln] *IL10*-[G(-1082)A] *COMT*-[Met108/158Val] *PTEN- *[(IVS4+109)ins/del5] *CCND1*-[Pro241Pro]	55.9%	0.046

*1 - prediction error.

## Discussion

In this study, we compared three methods, LRM and two partitioning methods, CART and MDR, for the detection of SNP-SNP interactions in a sample of 398 breast cancer cases and 372 population controls from Ontario. In addition to these three methods, we have applied a novel strategy to identify the strongest SNP-SNP interactions and assess their significance using cross-validation, re-sampling and permutation testing. Each approach models interactions differently; thus, they can be considered as a complementary set of methodologies to study SNP-SNP interactions in various disease models.

### Specificity of each method to identify SNP-SNP interactions

LRM can detect only low-order interactions as the model complexity increases with the order of interactions. This limitation of LRM is referred to as the curse of dimensionality [56]. A fully saturated model with numerous terms may be prone to unstable and biased estimates due to sparse data and multicolinearity. Furthermore, large sample theory underlying the test statistic may not hold. For certain SNP-SNP interactions, we found that the permutation distribution of the LRT did not closely match a chi-square distribution (results not shown), which justifies the use of a distribution-free test statistic. Alternative regression approaches, such as the logic model [57] multivariate adaptive regression splines (MARS) [58] or multivariate additive regression trees (MART) [59] may prove to be more efficient in this context. CART and MDR do not require or assume any specific parametric form for the relation between independent and dependent variables. Therefore, they might uncover SNP-SNP interactions that are missed by LRM. They can also deal with sparse and high-dimension data and can account for non-linear SNP-SNP interactions. An important feature of CART is the influence of the first split on the tree structure. In our analyses the only significant main effect, *XPD*-[Lys751Gln], appears in four out of the seven significant interactions. If there is no strong SNP main effect, the first variable selected to split the tree could change a lot due to small variation in the data. The resulting tree is interpreted as not being very stable. This problem is a concern in our data since all the main effects and low-order interactions are weak. Our strategy to generate multiple trees might reduce the influence of the SNP main effects in the resulting trees. Indeed, if an important pattern of interaction exists in the data, it is more likely to be detected by the use of multiple trees [59]. Methods such as patterning and recursive partitioning [60], random forests [61] or boosting [59] could reduce the influence of main effects on the results and will be further investigated. Two problems, however, are to assess the statistical significance of the resulting trees and to interpret the results. Sensitivity analyses may also help to assess the influence of each particular SNP on the pattern of SNP-SNP interactions. In order to evaluate the behavior of trees in the absence of SNPs with relatively stronger effects, we repeated the analysis without *XPD*-[Lys751Gln] and *CYP17*-[C(518)T]. Interestingly, the *COMT*-[Met108/158Val]*CCND1-[Pro241Pro] interaction, which was identified by MDR and LRM but not CART, was now also detected by CART analysis. MDR does not rely on binary splits as it performs a systematic search through all possible genotype combinations of the SNP variables, and thus could reveal more interactions than CART. The patterns of interactions suggested by MDR were indeed more diverse than those found by CART. However, the determination of statistical significance and interpretation of the results remain challenging with MDR. The interpretation can be facilitated by the use of entropy-based interaction dendrograms [51]. These methods allow MDR models to be decomposed into additive, independent, and synergistic effects, which greatly improves interpretation. The use of testing accuracy to select the best interactions can also be misleading in studies where the SNP predictive values are generally low. Limiting the SNP combinations to more plausible genetic models and selecting the important SNP-SNP interactions using a chi-square test statistic for contingency table instead of testing accuracy might provide useful improvements over the current MDR method. The pros and cons of each method are summarized in Table 4.

**Table 4:** Main characteristics of each approach used to model SNP-SNP interactions

Approach	Type of two-locus model detected	Pattern of complex interactions	Potential advantages	Potential limitations	Possible improvements
LRM	Logical AND models – multiplicative models	Can not be investigated	Easy to fit	Curse of dimensionality	Logic regression MARS*
CART	Conditional recessive or dominant models	Driven by SNP main effects and binary splits	Deals with sparse data Useful for risk characterization and prediction	Influence of main effects Redundancy	Random forest Boosting
MDR	All types	Diverse	Deals with sparse data Useful for risk characterization and prediction	Over-fitting Difficult to find best models Inefficient with large number of SNPs	Limit plausible genetic models Use test statistic

*Multivariate adaptive regression splines

### Application to genome-wide association studies

Genome-wide association studies using SNP arrays will remain particularly computationally intensive with the partitioning methods presented in this paper. Recently, Marchini *et al*. [62] suggested that such studies are computationally feasible, even when they include hundreds of thousands of loci. They also showed that even with a conservative correction for multiple testing, this strategy can be more powerful than traditional single-locus analyses. Their simulations, however, assumed some substantial marginal effects and relatively strong interaction effects that overcome the cost of the adjustment for multiple testing. It is not clear if the effects considered by the authors in their models are realistic or not. In our paper, we emphasize a two-stage approach to select the important interactions. This strategy has been previously advocated for large-scale association studies [63-66], in which the first stage is used to retain the most promising loci for further analyses, and can be adapted for methods such as CART and MDR. For example, Lunetta *et al*. [67] proposed to use random forests as a first-stage screening procedure to identify a small number of risk-associated SNPs, possibly interacting, from a large number of unassociated SNPs. A measure of importance for the low-order interactions could be developed for this purpose [68]. The interactions selected could then be validated in the second stage using the same approach or a CART analysis such as proposed in this paper. Some recent papers also discussed further extensions of MDR for genome-wide analysis of epistasis [69-72]. They also recommend an initial stage where the most promising interactions would be selected instead of performing an exhaustive search. They suggest two approaches: a 'filter' or a 'wrapper' strategy. The first approach, called Relief, provides an efficient filtering method accounting for the dependencies between variables (that is, SNP-SNP interactions) using a nearest neighbor algorithm. The second approach does not 'filter' (that is, discard some variables) but performs a stochastic search using a genetic programming algorithm to select variables that can interact in the absence of main effects. These are all very promising approaches. Finally, a possible selection of the important interactions could be based on prior information. Recently, Franke *et al*. [73] developed a Bayesian approach to reconstruct a functional human gene network that integrates information on genes and the functional relationships between them, based on data from multiple sources (the Kyoto Encyclopedia of Genes and Genomes, the Biomolecular Interaction Network Database, and so on). This method can help 'prioritize', in a first-stage analysis, candidate genes and gene-gene interactions associated with a specific disease, which could then be validated in an independent sample using the partitioning methods studied in our paper.

### Number and pattern of two-way SNP-SNP interactions

The number of significant two-way interactions was evaluated with all three methods. We found more significant two-way interactions with MDR (n = 4) compared to either CART (n = 2) or LRM (n = 3). For LRM, the limited number of interactions detected could be the result of the codominant effect assumed for each SNP, yielding a saturated model with numerous parameters to estimate. Assuming a dominant or recessive effect for each SNP could lead to a more parsimonious LRM and a more powerful test of interaction but with the price of a possible lack of fit. CART also detected only two interactions, and this could be due to a more stringent variable selection procedure than in LRM. However, the number of significant interactions should be interpreted with caution as each method might control the overall type-I error rate differently.

The pattern of interactions also varied according to the method used. Recently, Li and Reich [54] provided a complete enumeration and classification of two-locus disease models. Referring to this work, we tried to categorize the two-locus interactions suggested by each method. By its formulation, the LRM is more likely to detect interactions that act multiplicatively or additively on the disease risk [44]. These two-locus epistatic models are defined as 'logical AND models' and joint models [54]. With the 'logical AND models', for example, the dummy variable defining interaction will take the value 1 only if an individual carries one variant allele in each SNP and 0 otherwise. In CART, the binary splits tend to favor conditional recessive or conditional dominant epistatic models. Additive or multiplicative interaction effects might be more difficult to be captured by CART [55]. The interactions found by MDR suggest very complex two-locus epistatic models but most of them do not seem to have clear biological interpretation.

### Patterns of complex SNP-SNP interactions

The study of complex interactions was more exploratory in our study, although it revealed some interesting patterns worth mentioning. First, there was a high level of consistency in the patterns identified by both CART and MDR. The same important SNPs are contributing to the complex interactions and this is found with those two methods. We have determined that the SNPs more frequently detected by MDR and CART were more likely to be functional [41]. This suggests that our results do not occur only by chance but could reveal real biological links between genes within the same genetic pathways and between genes across different genetic pathways. The interactions detected by MDR seem more diverse and less influenced by the SNP main effects, but it is not clear whether the interactions detected are real or just false positives. Overall, our results suggest that the use of multiple statistical approaches (or an integrated approach) rather than a single methodology could be the best strategy to elucidate complex gene interactions that have generally very different patterns.

### Chance for false positive results

Our discovery of critical breast cancer associated SNP-SNP interactions involves two main steps: first, a data reduction technique that selects the most promising interactions based on cross-validation and second, an association testing method on a much smaller set of SNP-SNP interactions using permutation testing in the whole sample. In association testing of genetic variants, two-stage approaches are becoming popular, either as a cost-effective approach [63,64] or as a replication method [65,66]. In our study, it is more the latter aspect that was emphasized. Our first-stage analysis selected only the interactions that were replicated in an independent test set in order to have better consistency of the results. Indeed, the main purpose of our paper is to compare the specificity of each method to model SNP-SNP interactions and to identify the patterns of the selected interactions. No correction for multiple testing was applied so that the comparison of the methods could be performed with sufficient power. However, an important question remains as to whether the interactions identified by our study are really true or false positives. To get an answer to this question, an adjustment for multiple testing was performed in a single-stage analysis using only LRM and the FDR principle [53]. Using this multiple testing correction, four two-way interactions (including the two first interactions detected here by LRM) had FDR values lower than 5% [41]. Therefore, our new selection procedure might be slightly more conservative than a classical FDR adjustment, but further studies are needed to confirm this result. An alternative approach to correct for multiple testing could be to use permutation testing and assess the distribution of a maximum test statistic or minimum *P*-value after repeating the entire two-stage procedure n times (for example with n = 1,000) [74,75]. This method, which was applied to our data, also appears conservative compared to FDR adjustment but has the advantage of being very general. We are currently investigating this approach more specifically. Another adjustment could be to do a Bonferroni correction for the *K *tests performed at the second stage in the whole sample (*K *= 5 in our study for two-way interactions), to account for the number of interactions selected in the first stage analysis [65,66]. With this approach, only two two-way interactions would be considered significant (one with CART and one with MDR; Table 1).

Finally, to interpret our positive results, we should remember that our SNPs were selected on *a priori *evidence of being functionally important in the disease process [41]. Using a Bayesian approach, Wacholder *et al*. [76] showed how the probability of no true association between a genetic variant and a disease given a statistically significant result (that is, the false positive report probability (FPRP)) depends on the prior probability that the association is real and the statistical power of the test. Although the determination of a prior probability is quite challenging, selecting SNPs based on their functions clearly reduces the FPRP. Validation of our results in an independent dataset will strengthen the results we obtained and provide further insight into the role of interacting genes in breast cancer etiology.

### Biological interpretation of the results

Our study uses humans as a model organism and suggests the existence of interactions between the genes involved in various biological pathways related to breast cancer risk. A comprehensive study [77] has been published very recently demonstrating systematic epistatic interactions using yeast as a model organism. This study emphasized the co-dependency of genes from various functional categories to establish a phenotypic difference. Specifically, the authors showed that epistatic interactions could be organized into a network formed by functional modules and that interactions between functional modules are more likely to occur than within modules. In our study, the module could be thought of as a biological pathway, and the interactions between the SNPs would imply cross-talk between these pathways. Our results also suggest common interactions between SNPs within the same pathway and across different pathways, but further studies are needed to confirm this observation. As underlined by Moore and Williams [78], making biological interpretations from statistical models of epistasis is difficult to do for any method since we are trying to make inferences about biological processes at the cellular level in an individual from statistical summaries of variation in a population.

## Conclusion

There is growing evidence that gene interaction is not only possible but is probably ubiquitous in determining susceptibility to common human diseases [79,80]. The statistical approaches presented in this paper have the potential to assist in the identification of complex biological links among cancer processes involved in the development of breast cancer and could suggest new directions for the application and development of new statistical methods. This study not only provides insight into the analysis of the multi-genic nature of breast cancer, but also provides important information regarding how cell function relates to breast cancer development. We expect that these and other interactions involved in breast cancer etiology will one day be identified. This information will be used in clinics to identify individuals at increased risk of breast cancer and to develop preventive strategies.

## Competing interests

The author(s) declare that they have no competing interests.

## Authors' contributions

LB carried out the statistical analyses and wrote most of the manuscript. YW participated in statistical analysis and programming, especially the analyses with CART. She also contributed in the writing of some of the statistical sections of the manuscript. IR participated in some statistical analyses, especially with MDR. VO participated in design of the study, in data production and management, and manuscript preparation. JK participated in the epidemiological design of the study. ES participated in the statistical analyses, in particular, those involving the logistic regressions. HO participated in the design and coordination of the study, and manuscript preparation. All authors read and approved the final manuscript.

## Pre-publication history

The pre-publication history for this paper can be accessed here:



## Supplementary Material

Additional file 1 Positions of primers and probes used in the study in their appropriate accession numbers. Positions of primers and probes used in the study in their appropriate accession numbers. Click here for file

Additional file 2 Optimal MgCl_2 _concentrations and annealing temperatures for each SNP PCR. Optimal MgCl_2 _concentrations and annealing temperatures for each SNP PCR. Click here for file
